# Transcriptional Landscape of Glomerular Parietal Epithelial Cells

**DOI:** 10.1371/journal.pone.0105289

**Published:** 2014-08-15

**Authors:** Sina A. Gharib, Jeffrey W. Pippin, Takamoto Ohse, Scott G. Pickering, Ronald D. Krofft, Stuart J. Shankland

**Affiliations:** 1 Computational Medicine Core, Center for Lung Biology, Division of Pulmonary and Critical Care Medicine, University of Washington, Seattle, Washington, United States of America; 2 Division of Nephrology, University of Washington, Seattle, Washington, United States of America; 3 Division of Nephrology, University of Tokyo, Tokyo, Japan; University of Florida, United States of America

## Abstract

Very little is known about the function of glomerular parietal epithelial cells (PECs). In this study, we performed genome-wide expression analysis on PEC-enriched capsulated vs. PEC-deprived decapsulated rat glomeruli to determine the transcriptional state of PECs under normal conditions. We identified hundreds of differentially expressed genes that mapped to distinct biologic modules including development, tight junction, ion transport, and metabolic processes. Since developmental programs were highly enriched in PECs, we characterized several of their candidate members at the protein level. Collectively, our findings confirm that PECs are multifaceted cells and help define their diverse functional repertoire.

## Introduction

Parietal epithelial cells (PECs) are epithelial cells in the glomerulus that form a monolayer on the urinary side of Bowman’s capsule [1]. Unlike glomerular mesangial cells, endothelial cells, and their immediate neighbors podocytes, PECs have not been extensively studied. Recently however, this mysterious cell has attracted significant interest as some of its functions have been unraveled under physiological states [2], [3] For example, the tight junction proteins Claudin-1 and -2 have been identified in PECs that likely function as a secondary barrier to urinary filtrate [4]. Furthermore, PECs have been shown to likely serve as resident kidney stem cells in humans [5]–[8] as precursors for adult podocytes [9], and as transitional cells that express proteins of both PEC and podocyte origin in experimental disease states [10], [11].

While these studies have advanced our understanding of the important role played by PECs in health and disease, many properties of these cells remain undiscovered. We postulated that performing a comprehensive and unbiased assessment of PEC gene expression under normal, uninjured condition would shed light on its functional diversity and identify putative candidates mediating these biologic roles.

## Materials and Methods

### Glomerular isolation procedure

All glomerular isolations were performed at 4°C from individual male Sprague-Dawley rats (Charles River Laboratories, Wilmington, MA) per preparation (n = 3 animals). Rats were maintained at the animal care facilities at the University of Washington under a protocol approved by the Animal Care and Use Committee, according to Guide for the Care and Use of Laboratory Animals.

Obtaining glomeruli that contain Bowman’s capsule and the underlying PECs has traditionally been challenging using standard sieving or magnetic bead isolation techniques because these techniques typically obtain non-capsulated glomeruli that lack PECs [12], [13]. Accordingly, we developed a modified glomerular sieving protocol in order to generate glomerular fractions either enriched or devoid of PECs. The glomerular fractions were designated PEC-enriched (derived from capsulated glomeruli) or PEC-deprived (derived from decapsulated glomeruli) according to the size of the sieve used to isolate the glomeruli. After discarding medulla and papilla, minced cortices were passed through differential stainless steel sieves (VWR, West Chester, PA). In order to obtain capsulated glomeruli, minced cortices were first pressed through a 180 µm-sized mesh sieve and then washed through sequential sieves with sizes of 106 µm, 90 µm and 75 µm. The 90 µm and 75 µm sieves were used sequentially with ice-cold phosphate buffered saline to minimize the contamination of RNase. It was determined through preliminary studies that capsulated glomeruli enriched in PECs are much more adherent to the sieves than decapsulated glomeruli, and were therefore trapped by the 90 µm sieve (Figure S1A). In order to obtain decapsulated glomeruli deprived of PECs, the 90 µm sieve was removed and decapsulated glomeruli were collected on the 75 µm sieve (Figure S1B). Due to the adherent nature of isolated glomeruli, it was noted that many glomeruli suspended in PBS stick to plastic tubes, reducing the yield from an individual animal. Therefore, a solution of 0.1% Bovine Serum Albumin (BSA, Sigma-Aldrich, St. Louis, MO) in PBS was used instead and glass conical tubes (Kimble Glass, Inc., Vineland, NJ) coated with a silicon solution (Sigmacote, Sigma-Aldrich) were utilized following the sieving to reduce sticking and maximize yield. Glomeruli were centrifuged at 80 G at 4°C and quantification of glomeruli was performed in aliquots of the glomerular suspension by phase microscopy.

### RNA isolation

Pelleted glomeruli from individual rats were suspended in TRIzol reagent (Invitrogen) and disrupted by mechanical homogenizer (Tissuemiser, Thermo Fisher Scientific, Pittsburgh, PA), then frozen at –80°C as we have previously reported [14], [15] and according to the manufacturer’s instructions. RNA was re-suspended at a concentration of 25–500 ng/ml and the RNA integrity number (RIN) was assessed by a Bioanalyzer 2100 (Agilent Technologies, Santa Clara, CA). RNA samples with an RIN between 6 and 7 were utilized for microarray analysis.

### Microarray experiments and data analysis

High quality RNA samples were labeled and hybridized to Rat GeneChip 1.0ST microarrays following the manufacturer’s protocols (Affymetrix, Santa Clara, CA) at University of Washington’s Center for Expression Arrays. A total of six independent microarray experiments (n = 3 PEC-enriched capsulated, n = 3 PEC-deprived decapsulated) were performed. Background adjustment and quantile normalization of log-transformed intensities was performed using the RMA algorithm [16]. Detailed microarray experiment description, meeting Minimum Information About a Microarray Experiment (MIAME) requirements, has been deposited at Gene Expression Omnibus (http://www.ncbi.nlm.nih.gov/geo, GSE54085).

We applied multidimensional scaling using principal components analysis [17] to assess whether global variation in gene expression between glomerular cells derived from capsulated (PEC-enriched) and decapsulated (PEC-deprived) preparations would effectively discriminate between these two cell populations.

Differential gene expression between PEC-enriched and PEC-deprived samples was determined using Bayesian modeling of the parametric *t*-test [18] coupled with Benjamini-Hochberg adjustment of *P*-values for multiple hypothesis testing [19]. An adjusted *P*-value<0.01 was used to identify significant differential gene expression. We performed two dimensional hierarchical cluster analysis based on Euclidian distance to segregate differentially expressed genes and samples [17].

Functional enrichment analysis of differentially expressed genes was performed with Webgestalt program [20] and was based on Gene Ontology annotations [21]. Enrichment *P*-values were determined using Fisher’s exact test and corrected for multiple testing using Benjamini-Hochberg’s method with a significance *P*-value<0.01.

### Cell culture

Conditionally immortalized mouse parietal epithelial cells were generated as previously described [22]. In brief, H-2Kb-tsA58 mice, also called Immortomice (The Jackson Laboratory, Bar Harbor, ME) containing an interferon-γ inducible promoter for expression of thermo-sensitive SV40 large T antigen. PECs were isolated from Bowman’s capsule-containing glomerular outgrowths and characterization performed by immunostaining and western blot to cell specific-proteins as previously described [22]. Cells were initially cultured in growth permissive conditions on Primaria plastic plates (BD Biosciences, Bedford, MA) coated with collagen I (BD Biosciences) at 33°C with IFNγ (5 µL/10 mL media, BD Biosciences) in RPMI media (SH3025501 Fisher Scientific, Pittsburgh, PA) with 2% FBS (Gemni Bio-Products, West Sacramento, CA), penicillin/streptomycin (Sigma-Aldrich), sodium pyruvate (Fisher Scientific). Cells were then differentiated in growth-restricted conditions for at least 12 days by switching them to 37°C without IFNγ.

### PCR and Quantitative RT-PCR (qPCR)

PCR was performed on cultured murine PECs (mPECs) [22]. RNA was isolated from cultured mPECs and from frozen mouse kidney cortex using TRIzol reagent (Invitrogen) according to the manufacturer’s directions. 1 µg of RNA was used for cDNA synthesis using the Fermentas First Strand cDNA Synthesis Kit (Fermentas, Glen Burnie, MD). Reverse transcription PCR was carried out on a Biorad S1000 thermo cycler (BioRad, Hercules, CA) using the primers listed in Table S1 (Invitrogen). Amplified product was electrophoresed on a 2% agarose gel (Sigma Chemical). O’gene 100 bp DNA ladder Plus (Fermentas) was used to determine product size.

Similar to previously described studies [23], qPCR analysis was performed using QuantiTech Primer Assay and Rotor-Gene SYBR Green PCR Kits on a Corbett Rotor-Gene 6000 (Qiagen Inc. Valencia, CA). The same triplicate samples of total RNA from glomerular isolates was used for both microarray and qPCR experiments; in addition, we performed qPCR using total RNA from immortalized cultured mouse parietal epithelial cells [22] and podocytes [24]. To confirm candidate mRNAs, cDNA was synthesized using the QuantiTect Reverse Transcription Kit (Qiagen) Glyceraldehyde 3-phosphate dehydrogenase (GAPDH), was used as a reference gene to normalize the expression ratios. The cycle number (C_t_) values were averaged and the differences between the GAPDH C_t_ and the gene of interest C_t_ were calculated to determine the relative expression of the gene of interest using the 2^−ΔΔCt^ method [25]. The results are presented as fold change (capsulated vs. de-capsulated).

### Immunohistochemistry

To ensure that protein products of differentially expressed genes in capsulated glomeruli were indeed present in PECs, indirect immunoperoxidase staining was performed on renal biopsies from rats fixed in neutralized formalin and embedded in paraffin. Indirect immunoperoxidase staining was performed on 4-µm sections as previously reported [22] with minor modifications adapted to each primary antibody.

Paraffin was removed using Histoclear (National Diagnostics, Atlanta, GA) and sections were re-hydrated in ethanol. Antigen retrieval was achieved by boiling sections in 1 mM EDTA pH 8.0 (CDH6), 10 mM citric acid pH 7.0 (PAX8, ALDH1A1) or 10 mM citric acid pH 6.0 (FRAS1) [3], [4]. Endogenous peroxidase activity was quenched with 3% hydrogen peroxidase, non-specific protein binding was blocked with Background Buster (Accurate Chemical & Scientific Corporation, Westbury, NY) and endogenous biotin activity was quenched with the Avidin/biotin blocking kit (Vector Laboratories, Burlingame, CA). After blocking, tissue sections were incubated overnight at 4°C with the primary antibodies. The following antibodies were used: rabbit polyclonal anti-CDH6 1∶5000 (Abgent, San Diego, CA), rabbit polyclonal anti-PAX8 1∶250 (ProteinTech Group, Chicago, IL) rabbit polyclonal anti-ALDH1A1 1∶1000 (Epitomics, Burlingame, CA), and rabbit polyclonal anti-FRAS1 1∶2000 (Sigma-Aldrich). Either biotinylated-anti-rabbit IgG (Vector Laboratories) followed by R.T.U. Vectastain kit (Vector Laboratories) or PROMARK, Rabbit-on Rodent Polymer (HRP) (Biocare Medical, Concord, CA) were applied. Staining was visualized by precipitation of Diaminobenzidine (Sigma Chemical).

## Results

### Modified sieving protocol selectively isolates PEC-enriched glomerular cells *in vivo*

The percentage of capsulated (PEC enriched) vs. decapsulated (PEC deprived) as well as the percentage of tubules and glomerular number were determined to assure the quality of each preparation (Table 1). The 90 µm sieve preparations had a high percentage of capsulated and a lower percentage of decapsulated glomeruli, while the 75 µm preparations had a much lower percentage of capsulated and a higher percentage of decapsulated glomeruli. Both preparations contained between 0 to 12% tubular fragments and the total number of glomeruli isolated ranged from 8500 to 25,500. As expected, more RNA was obtained from the 90 µm fraction (PEC-enriched) compared with the 75 µm fraction (PEC-deprived) due to a higher number of glomeruli in the preparations and more cells within those glomeruli. All of the preparations generated had high RNA integrity numbers (RIN) between 6 and 7.

**Table 1:** Cellular characteristics of capsulated and decapsulated glomerular isolates.

Prep No.	Fraction	Capsulated Glomeruli% (PEC-enriched)	DecapsulatedGlomeruli %(PEC-deprived)	Tubules	Number ofGlomeruli	Total RNA	RIN*
1	90 µm	75%	16%	9%	25,500	31	6.9
2	90 µm	62%	28%	9%	14,500	23	6.4
3	90 µm	50%	43%	7%	20,250	21	6.4
4	75 µm	6%	82%	12%	10,750	11	6.3
5	75 µm	2%	98%	0%	8,500	5	6.5
6	75 µm	0%	100%	1%	20,400	7	7.0

*RIN: RNA integrity number.

### PEC-enriched isolates express distinct transcriptional signatures

**Figure 1:**
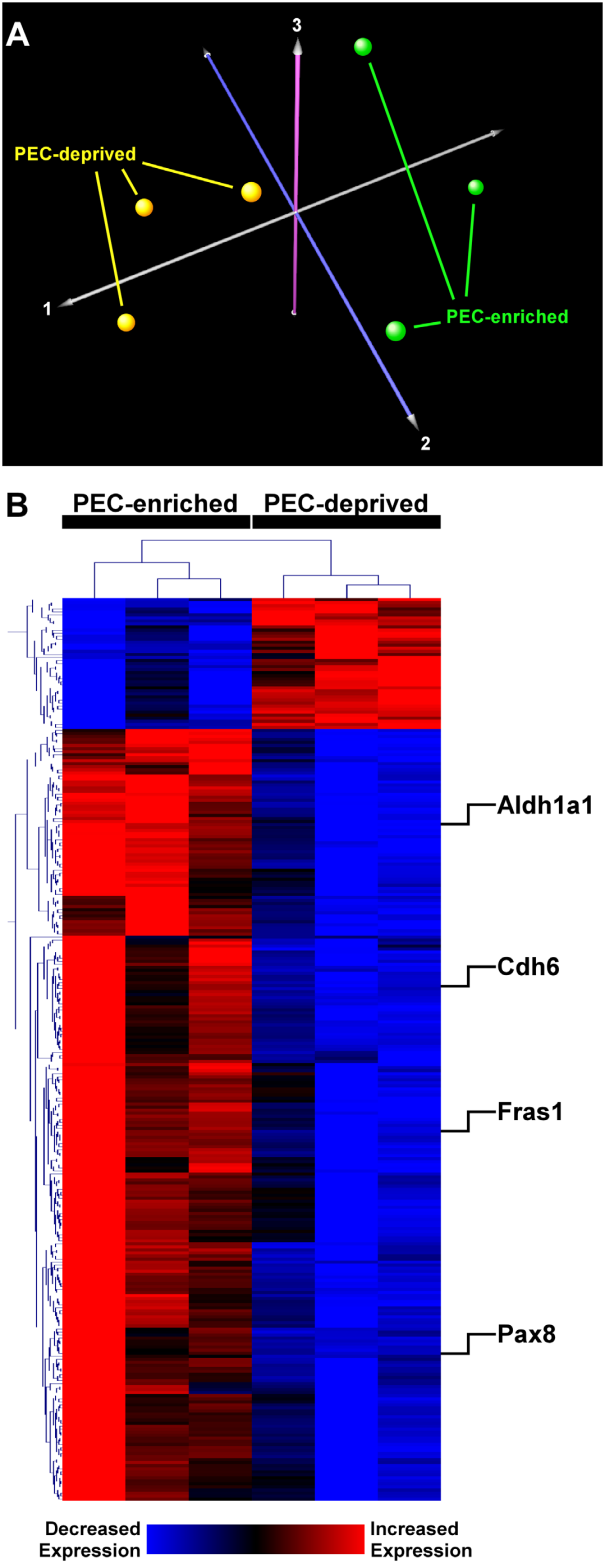
Transcriptional analysis of PEC-enriched and PEC-deprived glomerular isolates. (A) Principal components analysis of transcriptional signatures in PEC-enriched (n = 3) and PEC-deprived (n = 3) cells. Samples segregated into two groups (green, yellow) based on gene expression variability across all microarray transcripts. Each axis (labeled 1, 2, 3) accounts for a percentage of the observed gene expression variance. (B) Heatmap of differentially expressed genes between PEC-enriched and PEC-deprived showing up-regulated (red) and down-regulated (blue) gene expression patterns. Four representative candidate genes upregulated in PECs have been labeled. A complete list of differentially expressed genes is provided in Table S2.

Using principal components analysis, we assessed whether global variation in gene expression across all samples distinguished cell populations from each other and noted segregation between PEC-enriched and PEC-devoid samples (Figure 1A). This observation implies that these two glomerular cell populations have distinctly different transcriptional profiles. Next, we identified 334 probesets mapping to 276 unique, well-characterized genes that were differentially expressed between PEC-enriched and PEC-deprived populations (Figure 1B, Table S2). The majority of these genes (87%) were differentially upregulated in PEC-enriched glomerular isolates.

### Functional analysis reveals diverse biologic roles for PECs

**Figure 2:**
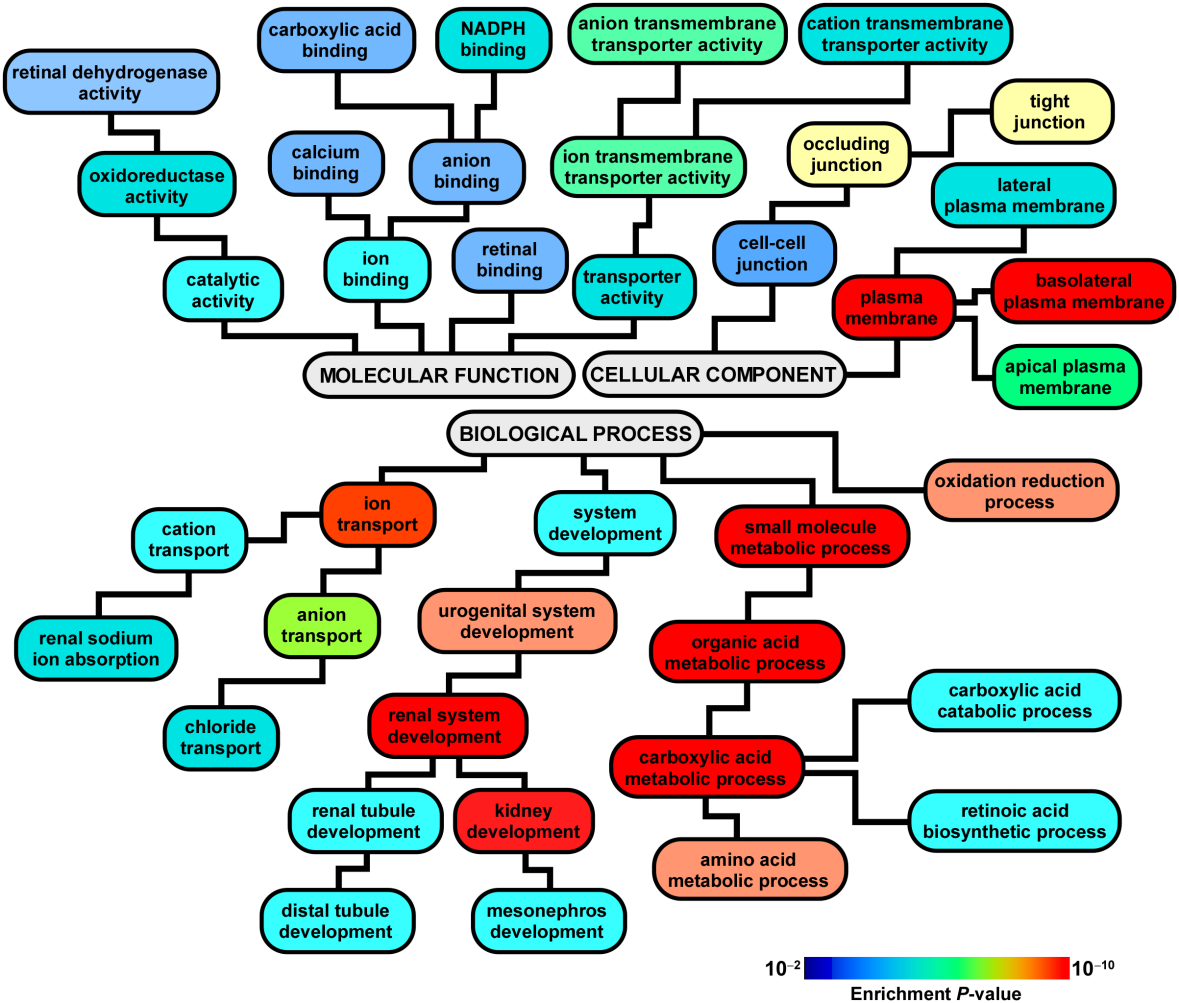
Functional analysis of differentially expressed genes. Significantly enriched Gene Ontology (GO) categories are depicted based on relational associations among GO annotations. Note the enrichment of multiple processes involved in development under the Biologic Process annotation. A complete list of over-represented GO categories is provided in Table S3.

Gene Ontology analysis of differentially expressed genes identified a broad repertoire of enriched functional annotations, highlighting the multifaceted biologic properties of PECs. Prominent overrepresented categories included those involved in development (in particular, “renal system development” with an enrichment *P*-value 8.6×10^–9^), ion transportation, metabolic processes, retinoic acid synthesis, tight junction, and oxidoreductase activity (Figure 2, complete list in Table S3). Enrichment of developmental pathways in PEC-enriched glomerular isolates was of particular interest given the putative role of PECs as resident kidney stem/progenitor cells [5]–[8]. Eighty differentially expressed genes mapped to developmental processes of which 71 were upregulated (Table S4). Several members of this module were further investigated as described below.

### Candidate PEC-associated genes are expressed in murine PEC cultures and at the protein level in rat and human glomeruli

We proceeded to validate our findings at several levels. Initially, we confirmed our microarray results for several candidate genes upregulated in the capsulated glomerular (>2-fold, adjusted *P*-value<0.01) using qPCR, including *Aldh1a1* (aldehyde dehydrogenase 1 family, member A1), *Cdh6* (cadherin 6), *Hnf1b* (HNF1 homeobox B), *Lad1* (ladinin 1), and *Wwc1* (WW and C2 domain containing 1) (Figure S2).

**Figure 3:**
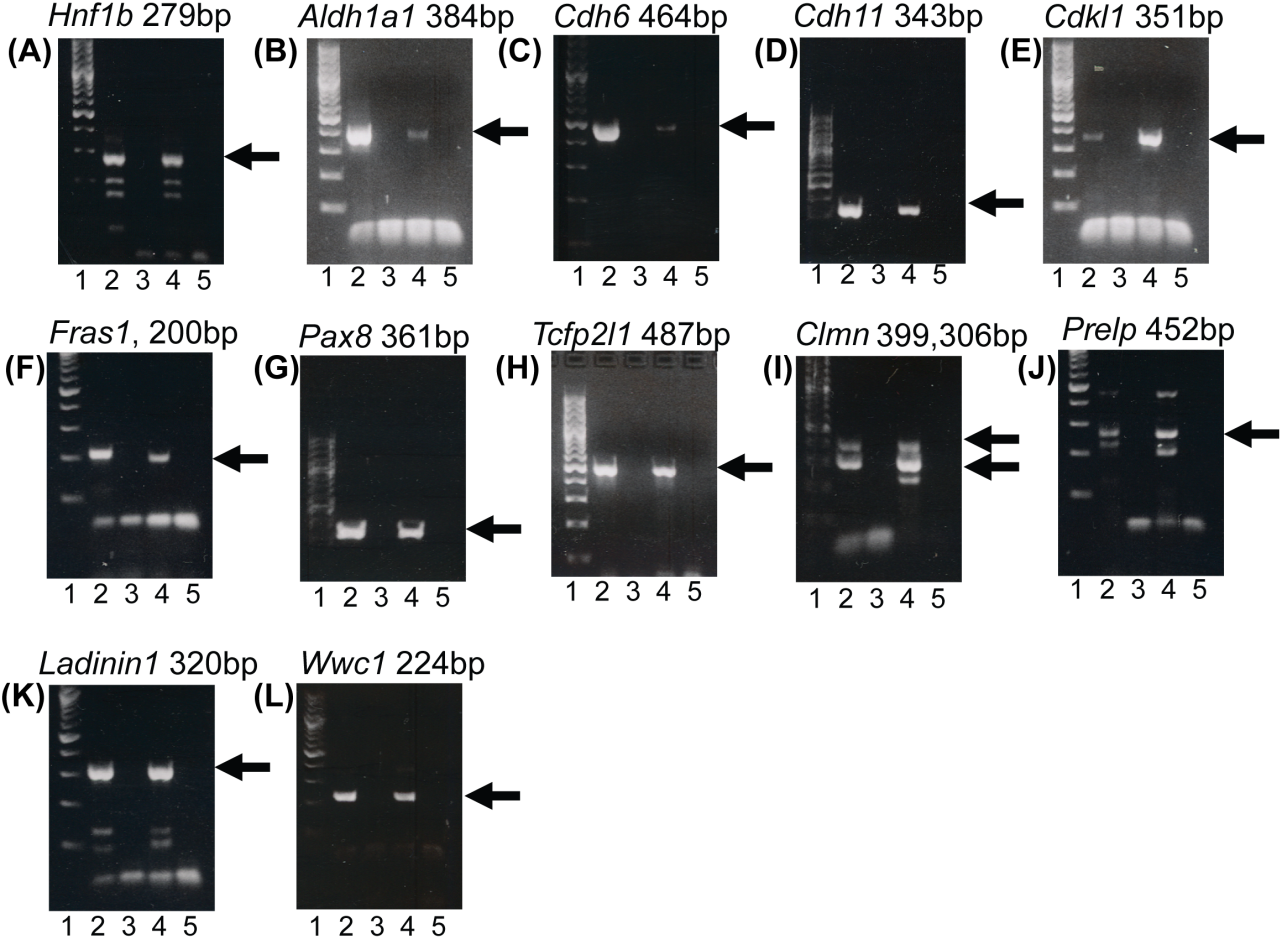
Confirmation of expression of select candidate genes from the microarray experiments in murine mPECs using PCR. Agarose gels were loaded as follows: lane 1 = DNA size marker, lane 2 = mPEC, lane 3 = mPEC no primer control, lane 4 = kidney cortex (positive control), lane 5 = kidney cortex no primer control. Arrows indicate positive bands: (A) *Hnf1b*; (B) *Aldh1a1*; (C) *Cdh6*; (D) *Cdh11*; (E) *Cdkl1*; (F) *Fras1*; (G) *Pax8*; (H) *Tfcp2l1*; (I) *Clmn*; (J) *Prelp*; (K) *Lad1*; (L) *Wwc1*.

Next, we proceeded to determine whether a larger set of PEC-associated genes was also expressed in cultured murine PECs (mPECs). Since cultured mPECs have minimal non-PEC cellular contaminants and are derived from mice, measuring the expression levels of our candidate genes would validate expression in a pure population of PECs and extend our findings from rat to mice, thereby expanding its relevance across species. Twelve differentially upregulated genes (>2-fold, adjusted *P*-value<0.01) in PEC-enriched samples were selected for confirmation in mPECs: *Aldh1a1*, *Cdh6*, *Hnf1b*, *Lad1*, *Wwc1, Clmn* (calmin), *Cdh11* (cadherin 11), *Cdkl1* (cyclin-dependent kinase-like 1), *Fras1* (Fraser syndrome 1), *Tfcp2l1* (transcription factor CP2-like 1), *Prelp* (proline/arginine-rich end leucine-rich repeat protein), and *Pax8* (paired box 8). Most of these genes were members of highly enriched Gene Ontology developmental processes (*Hnf1b, Aldh1a1, Cdh6, Cdh11, Cdkl1, Fras1*, *Pax8, Tfcp2l1, Clmn*), including “renal system development”. All twelve candidate genes were expressed at the mRNA level in cultures of pure mPECs (Figure 3). To assess specificity, we measured the relative expression levels of a representative PEC-associated candidate, *Pax8*, in cultures of mPECs versus murine podocytes using qPCR and found that this gene was up-regulated in mPECs 13-fold. However, it is important to note that *in vitro* culturing can dramatically change the transcriptional profile of primary cells [26] and may therefore not be a reliable platform for validating *in vivo* results.

**Figure 4:**
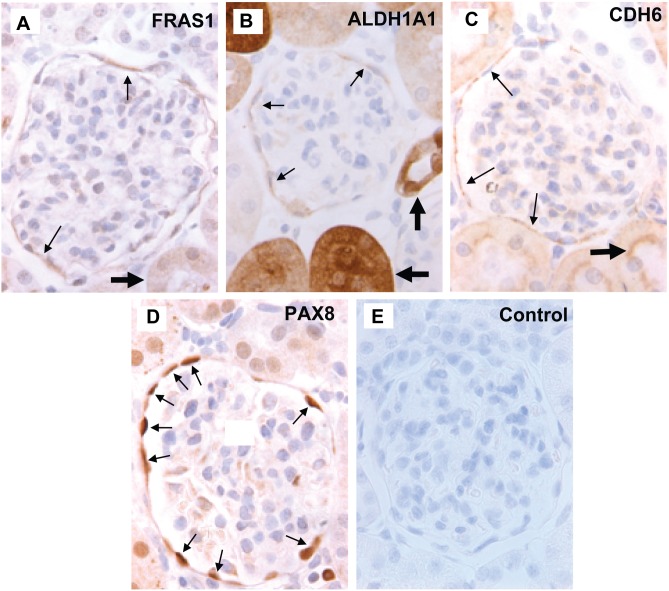
Protein expression of select developmental genes in rat kidney tissue. Panels A–D show representative images of immunohistochemistry on rat kidneys for: (A) FRAS1, cytoplasmic staining (brown) was present within PECs (thin arrows) as well as proximal tubules (thick arrow); (B) ALDH1A1, cytoplasmic staining (brown) was present within PECs (thin arrows) as well as proximal tubules and collecting duct (thick arrows); (C) CDH6, membrane staining (brown) was present in PECs (thin arrows) as well as the brush borders of proximal tubules (thick arrow); (D) PAX8, nuclear staining (brown) was present within PECs (thin arrows) as well as some tubules; (E) representative image of negative control with no immunostaining.

To validate protein expression of select development-associated candidate genes in rat kidney PECs, we obtained commercially available antibodies for ALDH1A1, CDH6, FRAS1 and PAX8. As shown in Figure 4, protein products from all four genes were detected in PECs as assessed by immunohistochemistry. Finally, to bring clinical insight to our findings, we queried the human protein atlas [27] and found that several of the PEC-associated candidates were expressed in human glomeruli, including HNF1B, ALDH1A1, CDH6, CLMN, LAD1, and WWC1 (Figure S3).

## Discussion

In this work we developed a novel and practical method to isolate PEC-enriched cells from rat glomeruli *in vivo*, and applied transcriptional profiling to systematically delineate potential biologic functions attributable to these cells under normal conditions.

Since the PEC is located within the glomerulus–a structure comprised of several other resident cells such as endothelium, mesangial cells and podocytes–it has proven challenging to separate PECs from other glomerular cells *in vivo*. While it is not difficult to obtain purified glomeruli from rat kidney, most isolated glomeruli lack Bowman’s capsule and are therefore devoid of PECs. Likewise, it is not possible to obtain capsulated glomeruli from mice even using the magnet bead procedure [13], because this technique requires a collagenase digestion step that removes Bowman’s capsule. In this project, we chose rat glomeruli because they do not require collagenase digestion. Modifications to standard sieving protocols were tested in our preliminary studies until a key observation was made when we attempted the ficoll gradient procedure to separate capsulated from decapsulated glomeruli based on size. When the diameter of isolated capsulated and decapsulated glomeruli were measured, we noted that decapsulated glomeruli were actually slightly larger than capsulated ones (data not shown). Additionally, capsulated glomeruli were much stickier than decapsulated glomeruli. The highly adherent property of PEC-enriched capsulated glomeruli trapped them on the larger pored 90 µm sieve, enabling their separation from the PEC-devoid decapsulated glomeruli.

To our knowledge no PEC-specific gene(s) has been identified to generate a PEC-specific reporter to sort labeled cells by FACS. The pioneering work by Appel and Moeller has substantially advanced the field using a truncated podocalyxin-reporter mouse to identify PECs [9]. However, scattered cells also label in the tubulointerstitium, albeit in low abundance. Kabgani and colleagues later isolated PEC outgrowths from glomeruli of mice based on this reporter system [28]. Gene expression analysis was then performed on PECs that had undergone six passages *ex vivo*. Despite significant differences between Kabgani’s methodology and our approach (e.g., mouse vs. rat, primary culture vs. *in vivo* harvesting), we confirmed the up-regulation of several PEC genes that they had reported, including *Claudin 1*, *Pax2*, and *Ladinin 1*. Guhr *et al* recently reported on the isolation of PECs employing a painstaking and laborious microscopic dissection method [29]. In contrast, our method is quick, efficient and uses standard isolation techniques.

In our study, the PEC-enriched and PEC-deprived samples were characterized by distinct differences in genome-wide transcription profiles, with many genes differentially expressed between the two populations (Figures 1 and 2). The unbiased nature of microarray experiments allowed us to assess relative enrichment of functional pathways in PEC-enriched samples. This analysis demonstrated that resident glomerular PECs under normal conditions possess multifaceted transcriptional programs, highlighting the diverse biologic roles played by these cells (Figure 2). One prominent enriched functional module was “tight junction”, comprised of several differentially upregulated genes including members of the Claudin family (*Cldn1*, *Cldn2*, *Cldn10*, *Cldn16*, *Cldn19*). Enrichment of processes involved in cell-cell junction was consistent with our previous report implicating PECs as a second glomerular barrier to urinary filtrate–a function mediated in part by the tight junction proteins CLDN1 and CLDN2 [4].

A key finding of our transcriptional analysis was the enrichment of multiple developmental processes in PECs, in particular, those mapping to renal development. This observation supports a potential role for PECs as possible resident kidney stem cells that retain fundamental developmental programs and are primed for activation during injury and repair. We further characterized several gene members of enriched developmental pathways at the protein level in rat kidney tissue.

*Fras1* is a gene mutated in Frasier Syndrome and encodes a basement membrane-associated protein important in renal development [30]. It is expressed in the branching ureteric bud of developing mouse kidney and on the basal aspect of glomerular podocytes [30]. We observed its expression at the BBM-facing aspect of rat PECs (Figure 4A, thin arrows), in the cytoplasm of the proximal tubule (Figure 4A, thick arrow), and weakly in glomerular podocytes.

*Aldh1a1* is an enzyme involved in the conversion of Vitamin A to retinoic acid. Several members of the aldehyde dehydrogenase enzymes play essential roles in renal development in both rat and mouse [31]. *Aldh1a1* has been found in all epithelia of the ureteric bud at day 14.5 of rat embryonic kidney and in the collecting ducts and proximal tubules by post-natal day 4 [32]. We confirmed these findings (Figure 4B, thick arrows) and further observed that *Aldh1a1* is also expressed in the cytoplasm of PECs (Figure 4B, thin arrows). Interestingly, Peired and co-workers recently showed endogenous *Aldh1a1* activity in a mouse model of FSGS in PECs [33].

*Cdh6* is a transmembrane glycoprotein and member of the cadherin family of calcium dependent cell-cell adhesion molecules [34]. CDH6 is expressed in human proximal tubules [35] and its targeted deletion leads to spontaneous tubule formation in mice [36]. Our results confirmed expression in the proximal tubule of rats (Figure 4C, thick arrow), as well as the basal surface of PECs (Figure 4C, thin arrows).

*Pax8* is a member of the paired box family of transcription factors that has been shown to play a critical role in kidney development [37], [38]. *Pax8* expression has previously been described in all segments of human renal tubules and in PECs [39]. Our finding demonstrates that this master regulator of renal development is expressed in rat PECs (Figure 4D, arrows) and is consistent with our previous report showing increased expression of *Pax8* during a murine model of glomerular disease [11].

There are several limitations in this study. Our sequential sieving procedure, while isolating enriched PECs, does not yield a pure population of this cell. In this context, the transcriptional contribution of other glomerular cells must be acknowledged. We used a limited number of animals to harvest capsulated and decapsulated glomeruli, but we were still able to detect consistent and significant transcriptional signals. Changes in gene expression do not necessarily imply alterations at the protein level, as has been demonstrated, for example, by Guhr and colleagues in rat PEC cultures [29]. Nevertheless, we confirmed the expression of several candidate proteins in PECs using PCR and immunostaining. We studied PECs under normal, uninjured condition to elucidate the basal biological roles of this cell in glomeruli. The transcriptional state of PECs is likely to be profoundly changed during injury and understanding the molecular consequences of such perturbations remains a future goal.

In summary, we developed a new, rapid isolation method to harvest capsulated glomeruli enriched in PECs and identified transcriptional signatures within this cell population that mapped to distinct functional categories. Several differentially expressed candidates were validated at the protein level using immunohistochemistry. Collectively, our findings implicate PECs as orchestrators of multiple biologic pathways in intact glomeruli and provide a roadmap for all to investigate their role in health and disease.

## Supporting Information

Figure S1 (A) Image of capsulated rat glomerulus obtained from the 90 µm pore fraction, enriched in PECs. (B) Image of a decapsulated rat glomerulus from the 75 µm pore fraction, deprived of PECs.
(PDF)

Figure S2 **qPCR confirmation of select differentially up-regulated candidate genes identified from microarray analysis of PEC-enriched capsulated (n = 3) vs. PEC-deprived decapsulated glomerular isolates (n = 3).**
(PDF)

Figure S3 **Immunohistochemical localization of six PEC-associated candidate proteins (HNF1B, ALDH1A1, CDH6, CLMN, LAD1, WWC1) in human glomeruli using the Human Protein Atlas database.** Arrows depict cytoplasmic or nuclear staining in PECs for each protein.
(PDF)

Table S1 **PCR Primers.**
(PDF)

Table S2 **List of differentially expressed, well-characterized genes between PEC-enriched and PEC-deprived glomerular isolates.** Genes are listed alphabetically.
(PDF)

Table S3 **List of significantly enriched Gene Ontology (GO) categories based on differentially expressed genes between PEC-enriched and PEC-deprived glomerular isolates.** GO categories have been organized into biologic process, cellular component and molecular function.
(PDF)

Table S4 **Alphabetical list of differentially expressed genes mapping to developmental processes.**
(PDF)
