# Supplementary material for: Transcriptional Landscape of Glomerular Parietal Epithelial Cells
Source: PLoS One. 2014 Aug 15;9(8):e105289. doi: 10.1371/journal.pone.0105289 (PMC4134297; doi:10.1371/journal.pone.0105289)

Supplemental Figure 1. (A) Image of capsulated rat glomerulus obtained from the 90μm pore fraction, enriched in PECs. (B) Image of a decapsulated rat glomerulus from the 75μm pore fraction, deprived of PECs.

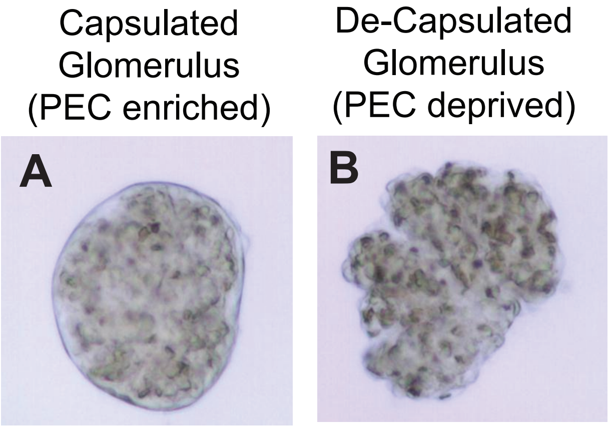

Supplement: Figure S1 — (A) Image of capsulated rat glomerulus obtained from the 90 µm pore fraction, enriched in PECs. (B) Image of a decapsulated rat glomerulus from the 75 µm pore fraction, deprived of PECs. (PDF) [file pone.0105289.s001.pdf]
