# Supplementary material for: Transcriptional Landscape of Glomerular Parietal Epithelial Cells
Source: PLoS One. 2014 Aug 15;9(8):e105289. doi: 10.1371/journal.pone.0105289 (PMC4134297; doi:10.1371/journal.pone.0105289)

Supplemental Figure 2. qPCR confirmation of select differentially up-regulated candidate genes identified from microarray analysis of PEC-enriched capsulated (n = 3) vs. PEC-deprived decapsulated glomerular isolates (n = 3).

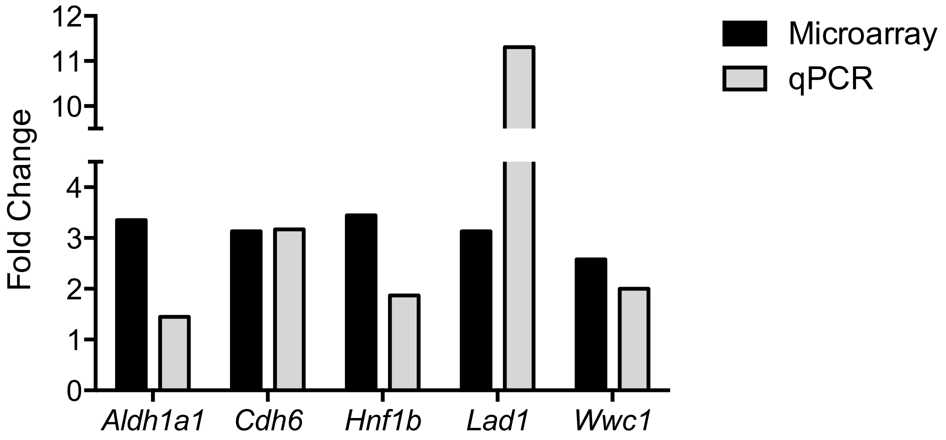

Supplement: Figure S2 — qPCR confirmation of select differentially up-regulated candidate genes identified from microarray analysis of PEC-enriched capsulated (n = 3) vs. PEC-deprived decapsulated glomerular isolates (n = 3). (PDF) [file pone.0105289.s002.pdf]
