# Supplementary material for: Transcriptional Landscape of Glomerular Parietal Epithelial Cells
Source: PLoS One. 2014 Aug 15;9(8):e105289. doi: 10.1371/journal.pone.0105289 (PMC4134297; doi:10.1371/journal.pone.0105289)

Supplemental Figure 3. Immunohistochemical localization of six PEC-associated candidate proteins (HNF1B, ALDH1A1, CDH6, CLMN, LAD1, WWC1) in human glomeruli using the Human Protein Atlas database. Arrows depict cytoplasmic or nuclear staining in PECs for each protein.

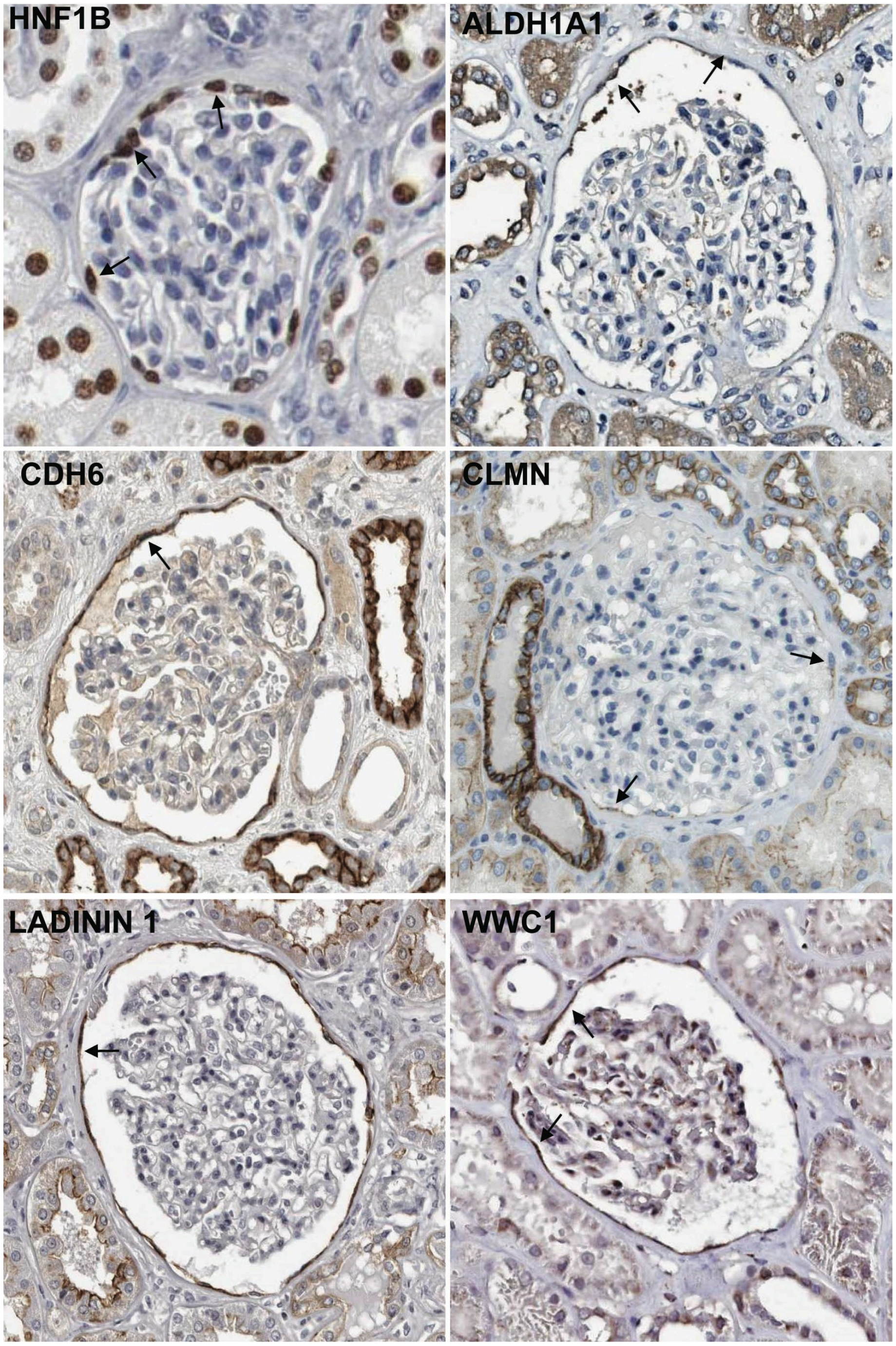

Supplement: Figure S3 — Immunohistochemical localization of six PEC-associated candidate proteins (HNF1B, ALDH1A1, CDH6, CLMN, LAD1, WWC1) in human glomeruli using the Human Protein Atlas database. Arrows depict cytoplasmic or nuclear staining in PECs for each protein. (PDF) [file pone.0105289.s003.pdf]
