# Supplementary material for: Transcriptional Landscape of Glomerular Parietal Epithelial Cells
Source: PLoS One. 2014 Aug 15;9(8):e105289. doi: 10.1371/journal.pone.0105289 (PMC4134297; doi:10.1371/journal.pone.0105289)
Supplement: Table S1 — PCR Primers. (PDF) [file pone.0105289.s004.pdf]

Supplementary Table 1. *PCR Primers*

Gene	*Forward*	*Reverse*
HNF1b	GAAAGCAACGGGAGATCCTC	CCTCCACTAAGGCCTCCCTC
Aldh1a1	CCTCTCACATGGATGTCGACAA	TCCCACTCTCAATGAGATCGAGTATT
CDH6	CTAGTGGCTTCCCAGCAAAG	CGTGACTTGGACCACAAATG
Cdh11	ACCAGATGTCTGTGTCAGA	GTCATCCTTGTCATCTGCA
Cdkl1	CAAACCAGCGAGGAAGACTC	AGGCTTTTGCCTTCTTCTCC
Fras1	CAGATGTCTTAGCTGTGCTGAGAAG	TTGGCAGACAGTGGCCTTGACGAAG
Pax8	CGTATTCCTCCTACAGTGAGG	CTGGAACTCCTGTCACTCACA
Tcfcp2l1	GTCAGGAGCTGGAGCAAAAC	ACCGCTGGCTTTCTTCTGTA
Calmin	CCTTGAAGAAAACTCCAGCG	AAGCAGTAAACGAGGAGCCA
Prelp	CTTCTAGCTGGCTCTCTGCT	CGGGAGCTCAGTGATGAAGTT
ladinin1	GCGACACCTCTTTGAGAAGG	GCAAGAGTCAAGGGTTCTGC
Wwc1	AGAGGTGGAGCAACTGGAGA	CAGGGCTACTGCCTGAAGAC
Gapdh	ACTGGTGTCTTCACCACCATGGAG	ACACGGAAGGCCATGCCAGTGAGC
