# Supplementary material for: Transcriptional Landscape of Glomerular Parietal Epithelial Cells
Source: PLoS One. 2014 Aug 15;9(8):e105289. doi: 10.1371/journal.pone.0105289 (PMC4134297; doi:10.1371/journal.pone.0105289)
Supplement: Table S2 — List of differentially expressed, well-characterized genes between PEC-enriched and PEC-deprived glomerular isolates. Genes are listed alphabetically. (PDF) [file pone.0105289.s005.pdf]

Supplementary Table 2. List of differentially expressed, well-characterized genes between PEC-enriched and PEC-deprived glomerular isolates. Genes are listed alphabetically.

Symbol	Entrez ID	Log_2[Fold Change]	Gene Name
Aadat	29416	1.325	aminoadipate aminotransferase
Acer2	313339	-0.821	alkaline ceramidase 2
Acmsd	171385	1.264	aminocarboxymuconate semialdehyde decarboxylase
Acsm2a	246263	0.944	acyl-CoA synthetase medium-chain family member 2A
Acss3	314800	0.773	acyl-CoA synthetase short-chain family member 3
Adamts15	300474	1.017	ADAM metallopeptidase with thrombospondin type 1 motif, 15
Agxt2	83784	1.035	alanine-glyoxylate aminotransferase 2
Akap12	83425	0.927	A kinase (PRKA) anchor protein 12
Akr1b10	296972	0.831	aldo-keto reductase family 1, member B10 (aldose reductase)
Aldh1a1	24188	1.744	aldehyde dehydrogenase 1 family, member A1
Aldh1a2	116676	0.925	aldehyde dehydrogenase 1 family, member A2
Aldh8a1	685750	1.105	aldehyde dehydrogenase 8 family, member A1
Aldob	24190	0.989	aldolase B, fructose-bisphosphate
Ank3	361833	0.797	ankyrin 3, node of Ranvier
Anpep	81641	1.104	alanyl (membrane) aminopeptidase
Antxr1	362393	1.242	anthrax toxin receptor 1
Ap1s3	367304	0.809	adaptor-related protein complex 1, sigma 3 subunit
Apol3	315108	-0.499	apolipoprotein L, 3
Apold1	444983	-0.703	apolipoprotein L domain containing 1
Aspa	79251	0.791	aspartoacylase
Ass1	25698	0.904	argininosuccinate synthase 1
Atp1b1	25650	1.267	ATPase, Na+/K+ transporting, beta 1 polypeptide
Atp2b4	29600	0.988	ATPase, Ca++ transporting, plasma membrane 4
Atp6v0a4	296981	1.059	ATPase, H+ transporting, lysosomal V0 subunit A4
Atp6v0d2	297932	1.074	ATPase, H+ transporting, lysosomal V0 subunit D2
Atp6v1b1	312488	1.172	ATPase, H transporting, lysosomal V1 subunit B1
B3galt5	288161	0.895	UDP-Gal:betaGlcNAc beta 1,3-galactosyltransferase, polypeptide 5
Baiap2l1	304282	0.835	BAI1-associated protein 2-like 1
Bnc2	298189	0.899	basonuclin 2
Bsnd	192675	0.819	Bartter syndrome, infantile, with sensorineural deafness (Barttin)
C5	362119	1.030	complement component 5
Ca2	54231	1.189	carbonic anhydrase 2
Cacna2d3	306243	1.178	calcium channel, voltage-dependent, alpha2/delta subunit 3
Calb1	83839	2.061	calbindin 1
Calcoco2	303479	-0.605	calcium binding and coiled-coil domain 2
Car12	363085	1.230	carbonic anyhydrase 12
Car15	288360	1.085	carbonic anhydrase 15
Caskin2	303678	-0.607	cask-interacting protein 2
Casr	24247	0.896	calcium-sensing receptor
Catsperg1	292767	0.690	cation channel, sperm-associated, gamma 1
Ccl2	24770	0.949	chemokine (C-C motif) ligand 2
Cd36	29184	-0.734	CD36 molecule (thrombospondin receptor)
Cdh11	84407	1.455	cadherin 11
Cdh16	307614	1.986	cadherin 16
Cdh6	25409	1.645	cadherin 6
Cdkl1	314198	1.619	cyclin-dependent kinase-like 1 (CDC2-related kinase)
Celsr2	83465	1.430	cadherin, EGF LAG seven-pass G-type receptor 2 (flamingo homolog, Drosophila)
Ces1e	29225	0.991	carboxylesterase 1E
Ces1f	100125372	1.158	carboxylesterase 1F
Chchd10	361824	0.913	coiled-coil-helix-coiled-coil-helix domain containing 10
Chst9	291770	0.969	carbohydrate (N-acetylgalactosamine 4-0) sulfotransferase 9
Clcnkb	79430	1.629	chloride channel Kb
Cldn1	65129	1.384	claudin 1
Cldn10	290485	1.378	claudin 10
Cldn16	155268	2.023	claudin 16
Cldn19	298487	0.685	claudin 19
Cldn2	300920	1.692	claudin 2
Clmn	299285	1.518	calmin

Cmbl	310201	1.063	carboxymethylenebutenolidase homolog (Pseudomonas)
Cmpk2	314004	-0.562	cytidine monophosphate (UMP-CMP) kinase 2, mitochondrial
Cobll1	311088	0.804	Cobl-like 1
Cox7b	303393	0.696	cytochrome c oxidase subunit VIIb
Cp	24268	1.008	ceruloplasmin (ferroxidase)
Cpm	314855	0.751	carboxypeptidase M
Cpne8	362988	-0.637	copine VIII
Cryz	362061	0.760	crystallin, zeta (quinone reductase)
Ctsc	25423	1.040	cathepsin C
Cyp4a1	50549	1.217	cytochrome P450, family 4, subfamily a, polypeptide 1
Cyp4a2	24306	1.320	cytochrome P450, family 4, subfamily a, polypeptide 2
Dao	114027	1.263	D-amino-acid oxidase
Dap	64322	0.624	death-associated protein
Dctd	290741	0.553	dCMP deaminase
Ddah1	64157	0.785	dimethylarginine dimethylaminohydrolase 1
Ddc	24311	0.959	dopa decarboxylase (aromatic L-amino acid decarboxylase)
Ddr1	25678	0.684	discoidin domain receptor tyrosine kinase 1
Defb1	83687	1.129	defensin beta 1
Degs2	314438	1.279	degenerative spermatocyte homolog 2, lipid desaturase (Drosophila)
Dlc1	58834	-0.585	deleted in liver cancer 1
Dll4	311332	-0.695	delta-like 4 (Drosophila)
Dnase1	25633	0.898	deoxyribonuclease I
Dsg2	307562	1.224	desmoglein 2
Dusp1	114856	-0.567	dual specificity phosphatase 1
Efemp1	305604	1.450	EGF-containing fibulin-like extracellular matrix protein 1
Efhd1	501181	1.063	EF-hand domain family, member D1
Egf	25313	2.094	epidermal growth factor
Egfl6	317470	1.469	EGF-like-domain, multiple 6
Ehd3	192249	-0.581	EH-domain containing 3
Ehf	295965	0.843	ets homologous factor
Emx2	499380	0.975	empty spiracles homeobox 2
Entpd2	64467	0.731	ectonucleoside triphosphate diphosphohydrolase 2
Epb41l3	116724	0.889	erythrocyte membrane protein band 4.1-like 3
Epcam	171577	1.332	epithelial cell adhesion molecule
Eps8	312812	0.871	epidermal growth factor receptor pathway substrate 8
Eps8l2	361674	0.829	EPS8-like 2
Erg	170909	-0.868	v-ets erythroblastosis virus E26 oncogene homolog (avian)
Errfi1	313729	0.944	ERBB receptor feedback inhibitor 1
Esrrg	360896	0.901	estrogen-related receptor gamma
Eya1	502935	0.935	eyes absent homolog 1 (Drosophila)
F2rl1	116677	0.786	coagulation factor II (thrombin) receptor-like 1
Fabp3	79131	1.101	fatty acid binding protein 3, muscle and heart
Fads2	83512	1.082	fatty acid desaturase 2
Fah	29383	0.781	fumarylacetoacetate hydrolase
Fam20c	304334	1.312	family with sequence similarity 20, member C
Fam212a	316001	-0.659	family with sequence similarity 212, member A
Fmo1	25256	0.942	flavin containing monooxygenase 1
Fras1	289486	1.604	Fraser syndrome 1
Fxyd2	29639	1.145	FXYD domain-containing ion transport regulator 2
Gabra1	29705	-0.950	gamma-aminobutyric acid (GABA) A receptor, alpha 1
Gadd45a	25112	0.851	growth arrest and DNA-damage-inducible, alpha
Gca	295647	1.542	grancalcin
Ggct	362368	0.766	gamma-glutamyl cyclotransferase
Ggct	362368	0.766	gamma-glutamyl cyclotransferase
Gimap6	297076	-0.690	GTPase, IMAP family member 6
Gimap8	500112	-0.589	GTPase, IMAP family member 8
Gimap9	493865	-0.706	GTPase, IMAP family member 9
Glb1l2	503194	0.995	galactosidase, beta 1-like 2
Glyat	293779	1.157	glycine-N-acyltransferase
Gpc3	25236	0.778	glypican 3
Gpm6a	306439	0.792	glycoprotein m6a
Gpr146	498153	-0.671	G protein-coupled receptor 146

Gpr39	288995	0.876	G protein-coupled receptor 39
Gpr56	260326	1.101	G protein-coupled receptor 56
Gprc5a	312790	0.800	G protein-coupled receptor, family C, group 5, member A
Gprc5c	287805	0.829	G protein-coupled receptor, family C, group 5, member C
Grap	363616	-0.578	GRB2-related adaptor protein
Grb7	84427	0.677	growth factor receptor bound protein 7
Grhpr	680021	0.898	glyoxylate reductase/hydroxypyruvate reductase
Gsta4	300850	1.173	glutathione S-transferase alpha 4
Gsta4	300850	0.988	glutathione S-transferase alpha 4
Gsta4	300850	0.766	glutathione S-transferase alpha 4
Hao2	84029	0.976	hydroxyacid oxidase 2 (long chain)
Heyl	313575	0.781	hairy/enhancer-of-split related with YRPW motif-like
Hlx	364069	-0.735	H2.0-like homeobox
Hnf1b	25640	1.786	HNF1 homeobox B
Hnf4a	25735	0.918	hepatocyte nuclear factor 4, alpha
Hoga1	293949	0.660	4-hydroxy-2-oxoglutarate aldolase 1
Hpd	29531	0.994	4-hydroxyphenylpyruvate dioxygenase
Hrsp12	65151	1.147	heat-responsive protein 12
Hsd11b1	25116	0.915	hydroxysteroid 11-beta dehydrogenase 1
Hyal1	367166	-0.632	hyaluronoglucosaminidase 1
Idi1	89784	0.680	isopentenyl-diphosphate delta isomerase 1
Impa2	282636	0.890	inositol (myo)-1(or 4)-monophosphatase 2
Inadl2	140581	0.762	InaD-like 2 (Drosophila)
Inmt	368066	1.767	indolethylamine N-methyltransferase
Itgb6	311061	1.362	integrin, beta 6
Kcnj1	24521	1.384	potassium inwardly-rectifying channel, subfamily J, member 1
Kcnj15	170847	0.931	potassium inwardly-rectifying channel, subfamily J, member 15
Kcnj16	29719	1.262	potassium inwardly-rectifying channel, subfamily J, member 16
Kcnk1	59324	1.249	potassium channel, subfamily K, member 1
Kif12	313254	1.326	kinesin family member 12
Kl	83504	1.003	Klotho
Klhdc8a	305096	1.364	kelch domain containing 8A
Klhl29	298867	0.958	kelch-like 29 (Drosophila)
Klk1	24594	1.818	kallikrein 1
Klk1c7	24523	0.915	kallikrein 1-related peptidase C7
Krt8	25626	1.254	keratin 8
Krt8	25626	0.784	keratin 8
L1cam	50687	1.251	L1 cell adhesion molecule
Lad1	313325	1.647	ladinin 1
Lad1	313325	1.122	ladinin 1
Lcn2	170496	1.326	lipocalin 2
Lipe	25330	-0.676	lipase, hormone sensitive
Lox	24914	1.126	lysyl oxidase
Ly6b	246138	2.321	lymphocyte antigen 6 complex, locus B
Lypd6b	362133	0.822	LY6/PLAUR domain containing 6B
Mal2	362911	1.515	mal, T-cell differentiation protein 2
Map7	293016	0.822	microtubule-associated protein 7
Mboat2	313997	0.798	membrane bound O-acyltransferase domain containing 2
Mcf2l	117020	-0.663	MCF.2 cell line derived transforming sequence-like
Me3	361602	0.827	malic enzyme 3, NADP(+)-dependent, mitochondrial
Mfap3l	306424	1.406	microfibrillar-associated protein 3-like
MGC112715	690899	-0.670	hypothetical protein LOC690899
Miox	252899	1.113	myo-inositol oxygenase
Mme	24590	1.139	membrane metallo-endopeptidase
Myh11	24582	0.856	myosin, heavy chain 11, smooth muscle
Myo5b	25132	1.119	myosin Vb
Nav2	171563	0.816	neuron navigator 2
Ncs1	65153	0.956	neuronal calcium sensor 1
Ndrg1	299923	0.949	N-myc downstream regulated 1
Ndrg2	171114	0.696	N-myc downstream regulated gene 2
Nos1	24598	0.823	nitric oxide synthase 1, neuronal
Obfc2a	363227	0.742	oligonucleotide/oligosaccharide-binding fold containing 2A

Ocln	83497	1.163	occludin
Osbpl3	362360	0.936	oxysterol binding protein-like 3
Padi2	29511	0.971	peptidyl arginine deiminase, type II
Pah	24616	1.133	phenylalanine hydroxylase
Pamr1	311252	1.138	peptidase domain containing associated with muscle regeneration 1
Paqr5	315741	0.993	progestin and adipoQ receptor family member V
Pax2	293992	0.697	paired box 2
Pax8	81819	1.552	paired box 8
Pbld	171564	1.089	phenazine biosynthesis-like protein domain containing
Pcbd1	29700	0.958	pterin-4 alpha-carbinolamine dehydratase/dimerization cofactor of hepatocyte nuclear factor 1 alpha
Pdgfc	79429	1.119	platelet derived growth factor C
Pdzk1	65144	1.104	PDZ domain containing 1
Pdzk1ip1	81916	1.206	PDZK1 interacting protein 1
Pfn2	81531	0.650	profilin 2
Phyh	114209	0.855	phytanoyl-CoA 2-hydroxylase
Pigr	25046	0.873	polymeric immunoglobulin receptor
Plau	25619	1.654	plasminogen activator, urokinase
Plekha6	360842	0.697	pleckstrin homology domain containing, family A member 6
Pls1	315926	0.944	plastin 1
Pon3	312086	1.117	paraoxonase 3
Ppargc1a	83516	0.696	peroxisome proliferator-activated receptor gamma, coactivator 1 alpha
Ppp1r16b	680616	-0.618	protein phosphatase 1, regulatory subunit 16B
Ppp1r1a	58977	1.111	protein phosphatase 1, regulatory (inhibitor) subunit 1A
Prelp	84400	1.529	proline/arginine-rich end leucine-rich repeat protein
Prkcz	25522	0.815	protein kinase C, zeta
Prosapip1	280670	0.736	ProSAPiP1 protein
Prss8	192107	1.111	protease, serine, 8
Ptafr	58949	-0.621	platelet-activating factor receptor
Pter	63852	0.791	phosphotriesterase related
Ptprf	360406	1.040	protein tyrosine phosphatase, receptor type, F
Rab17	503269	0.925	RAB17, member RAS oncogene family
Rab3ip	29885	0.745	RAB3A interacting protein (rabin3)
Ramp2	58966	-0.645	receptor (G protein-coupled) activity modifying protein 2
Ramp3	56820	-0.711	receptor (G protein-coupled) activity modifying protein 3
Rap1gap2	303298	0.725	RAP1 GTPase activating protein 2
Rbm47	305340	1.225	RNA binding motif protein 47
Rbp1	25056	1.171	retinol binding protein 1, cellular
Ren	24715	1.544	renin
Rhcg	293048	0.849	Rh family, C glycoprotein
Rimklb	362428	0.939	ribosomal modification protein rimK-like family member B
Rimklb	362428	0.933	ribosomal modification protein rimK-like family member B
Rin2	311494	-0.568	Ras and Rab interactor 2
Rragd	297960	0.791	Ras-related GTP binding D
S1pr1	29733	-0.642	sphingosine-1-phosphate receptor 1
Scube3	294297	0.686	signal peptide, CUB domain, EGF-like 3
Serf1	502503	0.760	small EDRK-rich factor 1
Sgpp2	301543	0.846	sphingosine-1-phosphate phosphatase 2
Sh2d3c	362111	-0.627	SH2 domain containing 3C
Slc12a1	25065	2.566	solute carrier family 12 (sodium/potassium/chloride transporters), member 1
Slc12a3	54300	1.969	solute carrier family 12 (sodium/chloride transporters), member 3
Slc13a3	64846	1.031	solute carrier family 13 (sodium-dependent dicarboxylate transporter), member 3
Slc16a12	309525	1.140	solute carrier family 16, member 12 (monocarboxylic acid transporter 12)
Slc16a14	316578	0.804	solute carrier family 16, member 14 (monocarboxylic acid transporter 14)
Slc16a7	29735	0.983	solute carrier family 16, member 7 (monocarboxylic acid transporter 2)
Slc17a4	679784	1.251	solute carrier family 17 (sodium phosphate), member 4
Slc1a1	25550	1.085	solute carrier family 1, member 1
Slc22a25	192273	1.080	solute carrier family 22, member 25
Slc25a23	301113	0.893	solute carrier family 25 (mitochondrial carrier phosphate carrier), member 23
Slc26a7	297910	0.782	solute carrier family 26, member 7
Slc3a1	29484	1.175	solute carrier family 3, member 1
Slc43a2	287532	0.936	solute carrier family 43, member 2
Slc43a3	311170	-1.087	solute carrier family 43, member 3

Slc44a4	294255	1.044	solute carrier family 44, member 4
Slc4a1	24779	0.758	solute carrier family 4 (anion exchanger), member 1
Slc4a4	84484	1.020	solute carrier family 4, sodium bicarbonate cotransporter, member 4
Slc4a9	266612	0.938	solute carrier family 4, sodium bicarbonate cotransporter, member 9
Slc7a7	83509	1.012	solute carrier family 7 (amino acid transporter light chain, y+L system), member 7
Slit2	360272	1.160	slit homolog 2 (Drosophila)
Slit3	83467	0.940	slit homolog 3 (Drosophila)
Smtnl2	679629	0.993	smoothelin-like 2
Snrk	170837	-0.560	SNF related kinase
Sord	24788	0.935	sorbitol dehydrogenase
Sorl1	300652	0.767	sortilin-related receptor, LDLR class A repeats-containing
Sostdc1	266803	1.403	sclerostin domain containing 1
Spink1	266602	1.274	serine peptidase inhibitor, Kazal type 1
Spint1	311331	0.767	serine peptidase inhibitor, Kunitz type 1
Spint2	292770	0.830	serine peptidase inhibitor, Kunitz type, 2
Spta1	289257	0.976	spectrin, alpha, erythrocytic 1 (elliptocytosis 2)
St14	114093	0.721	suppression of tumorigenicity 14 (colon carcinoma)
Stk32b	305431	0.792	serine/threonine kinase 32B
Sytl2	361604	0.874	synaptotagmin-like 2
Tagln	25123	0.738	transgelin
Tfcp2l1	304741	1.386	transcription factor CP2-like 1
Tfrc	64678	0.754	transferrin receptor
Tinag	300846	1.206	tubulointerstitial nephritis antigen
Tmem171	293634	0.756	transmembrane protein 171
Tmem2	309400	-0.665	transmembrane protein 2
Tmem27	57395	1.324	transmembrane protein 27
Tmem72	362424	0.909	transmembrane protein 72
Tmprss2	156435	1.280	transmembrane protease, serine 2
Umod	25128	2.605	uromodulin
Upp1	289801	-0.573	uridine phosphorylase 1
Wwc1	303039	1.366	WW and C2 domain containing 1
Xpnpep2	117522	1.082	X-prolyl aminopeptidase (aminopeptidase P) 2, membrane-bound
