# Supplementary material for: Transcriptional Landscape of Glomerular Parietal Epithelial Cells
Source: PLoS One. 2014 Aug 15;9(8):e105289. doi: 10.1371/journal.pone.0105289 (PMC4134297; doi:10.1371/journal.pone.0105289)
Supplement: Table S3 — List of significantly enriched Gene Ontology (GO) categories based on differentially expressed genes between PEC-enriched and PEC-deprived glomerular isolates. GO categories have been organized into biologic process, cellular component and molecular function. (PDF) [file pone.0105289.s006.pdf]

Supplementary Table 3. List of significantly enriched Gene Ontology (GO) categories based on differentially expressed genes between PEC-enriched and PEC-deprived glomerular isolates. GO categories have been organized into biologic process, cellular component and molecular function.

GO Annotation	GO Category	Fold Enrichment	P-value	Adjusted P-value
biological process	carboxylic acid metabolic process	3.35	3.68E-11	4.29E-08
biological process	small molecule metabolic process	2.30	4.95E-11	4.29E-08
biological process	organic acid metabolic process	3.18	9.46E-11	5.47E-08
biological process	oxoacid metabolic process	3.16	2.03E-10	8.81E-08
biological process	renal system development	4.73	8.61E-09	2.99E-06
biological process	kidney development	5.10	1.53E-08	4.29E-06
biological process	ion transport	2.61	1.73E-08	4.29E-06
biological process	urogenital system development	4.09	9.92E-08	1.91E-05
biological process	oxidation-reduction process	3.29	9.11E-08	1.91E-05
biological process	alpha-amino acid metabolic process	5.45	2.83E-07	4.91E-05
biological process	metanephric distal convoluted tubule development	45.75	4.49E-07	6.49E-05
biological process	distal convoluted tubule development	45.75	4.49E-07	6.49E-05
biological process	small molecule catabolic process	4.36	8.44E-07	9.75E-05
biological process	single-organism catabolic process	4.34	8.99E-07	9.75E-05
biological process	cellular amino acid metabolic process	4.36	8.44E-07	9.75E-05
biological process	aromatic amino acid family metabolic process	17.16	8.43E-07	9.75E-05
biological process	anion transport	3.63	1.26E-06	1.00E-04
biological process	response to organic substance	1.88	1.55E-06	1.00E-04
biological process	small molecule biosynthetic process	3.31	2.83E-06	2.00E-04
biological process	organonitrogen compound metabolic process	2.05	2.94E-06	2.00E-04
biological process	metanephric distal tubule development	32.68	3.06E-06	2.00E-04
biological process	L-phenylalanine metabolic process	32.68	3.06E-06	2.00E-04
biological process	monocarboxylic acid metabolic process	3.33	2.47E-06	2.00E-04
biological process	single-organism biosynthetic process	3.21	4.35E-06	3.00E-04
biological process	ion transmembrane transport	3.01	1.15E-05	8.00E-04
biological process	cation transport	2.46	1.39E-05	9.00E-04
biological process	organic acid catabolic process	4.37	1.99E-05	1.10E-03
biological process	retinoic acid biosynthetic process	22.88	1.76E-05	1.10E-03
biological process	diterpenoid biosynthetic process	22.88	1.76E-05	1.10E-03
biological process	carboxylic acid catabolic process	4.37	1.99E-05	1.10E-03
biological process	system development	1.52	2.03E-05	1.10E-03
biological process	renal sodium ion absorption	42.89	2.08E-05	1.10E-03
biological process	nephron tubule development	9.80	2.84E-05	1.40E-03
biological process	distal tubule development	20.80	2.73E-05	1.40E-03
biological process	aromatic amino acid family catabolic process	20.80	2.73E-05	1.40E-03
biological process	renal tubule development	9.53	3.35E-05	1.50E-03
biological process	mesonephros development	13.00	3.23E-05	1.50E-03
biological process	chloride transport	7.70	3.12E-05	1.50E-03
biological process	vitamin A biosynthetic process	34.31	5.14E-05	2.00E-03
biological process	terpenoid biosynthetic process	17.60	5.75E-05	2.00E-03
cellular component	cell periphery	1.99	2.98E-13	2.89E-11
cellular component	plasma membrane	2.02	2.30E-13	2.89E-11
cellular component	plasma membrane part	2.51	2.51E-10	1.62E-08
cellular component	basolateral plasma membrane	6.19	7.14E-10	3.46E-08
cellular component	occluding junction	7.94	1.22E-07	3.94E-06
cellular component	tight junction	7.94	1.22E-07	3.94E-06
cellular component	apical part of cell	3.88	2.44E-07	6.76E-06
cellular component	apical junction complex	6.55	8.95E-07	2.17E-05
cellular component	apical plasma membrane	3.87	8.35E-06	2.00E-04
cellular component	extracellular region part	2.15	4.51E-05	9.00E-04
cellular component	cell-cell junction	3.12	2.00E-04	3.50E-03
cellular component	membrane	1.23	5.00E-04	6.10E-03

cellular component	recycling endosome membrane	10.50	5.00E-04	6.10E-03
cellular component	cell	1.11	5.00E-04	6.10E-03
cellular component	cell part	1.11	5.00E-04	6.10E-03
cellular component	lateral plasma membrane	8.02	4.00E-04	6.10E-03
cellular component	vacuolar proton-transporting V-type ATPase complex	15.75	8.00E-04	9.10E-03
molecular function	cofactor binding	3.96	1.45E-06	3.00E-04
molecular function	retinal dehydrogenase activity	33.44	2.79E-06	3.00E-04
molecular function	carboxylic acid binding	4.40	3.71E-06	3.00E-04
molecular function	NADP binding	9.99	5.31E-06	3.00E-04
molecular function	anion transmembrane transporter activity	4.03	5.04E-06	3.00E-04
molecular function	ion transmembrane transporter activity	2.44	7.54E-06	4.00E-04
molecular function	catalytic activity	1.39	2.20E-05	8.00E-04
molecular function	ion binding	1.42	2.12E-05	8.00E-04
molecular function	substrate-specific transporter activity	2.20	2.05E-05	8.00E-04
molecular function	substrate-specific transmembrane transporter activity	2.26	3.30E-05	1.00E-03
molecular function	transmembrane transporter activity	2.18	4.41E-05	1.20E-03
molecular function	transporter activity	2.03	4.57E-05	1.20E-03
molecular function	anion:cation symporter activity	11.70	5.60E-05	1.40E-03
molecular function	coenzyme binding	3.84	7.21E-05	1.50E-03
molecular function	oxidoreductase activity	2.29	6.94E-05	1.50E-03
molecular function	inorganic cation transmembrane transporter activity	2.63	1.00E-04	2.00E-03
molecular function	chloride transmembrane transporter activity	5.85	2.00E-04	3.70E-03
molecular function	phenylalanine 4-monooxygenase activity	58.52	3.00E-04	5.30E-03
molecular function	inorganic anion transmembrane transporter activity	4.46	4.00E-04	6.70E-03
molecular function	identical protein binding	1.98	5.00E-04	6.90E-03
molecular function	cation transmembrane transporter activity	2.29	5.00E-04	6.90E-03
molecular function	vitamin binding	4.33	5.00E-04	6.90E-03
molecular function	NADPH binding	17.56	5.00E-04	6.90E-03
molecular function	metal ion transmembrane transporter activity	2.63	6.00E-04	7.60E-03
molecular function	monovalent inorganic cation transmembrane transporter activity	2.77	6.00E-04	7.60E-03
molecular function	long-chain fatty acid binding	15.96	7.00E-04	7.70E-03
molecular function	calcium ion binding	2.29	7.00E-04	7.70E-03
molecular function	anion binding	1.56	7.00E-04	7.70E-03
molecular function	retinal binding	15.96	7.00E-04	7.70E-03
