# Supplementary material for: Transcriptional Landscape of Glomerular Parietal Epithelial Cells
Source: PLoS One. 2014 Aug 15;9(8):e105289. doi: 10.1371/journal.pone.0105289 (PMC4134297; doi:10.1371/journal.pone.0105289)
Supplement: Table S4 — Alphabetical list of differentially expressed genes mapping to developmental processes. (PDF) [file pone.0105289.s007.pdf]

Supplementary Table 4. Alphabetical list of differentially expressed genes mapping to developmental processes.

Symbol	Entrez ID	Log_2[Fold Change]	Gene Name
Aldh1a1	24188	1.744	aldehyde dehydrogenase 1 family, member A1
Aldh1a2	116676	0.925	aldehyde dehydrogenase 1 family, member A2
Aldob	24190	0.989	aldolase B, fructose-bisphosphate
Ank3	361833	0.797	ankyrin 3, node of Ranvier
Anpep	81641	1.104	alanyl (membrane) aminopeptidase
Apold1	444983	-0.703	apolipoprotein L domain containing 1
Aspa	79251	0.791	aspartoacylase
Ass1	25698	0.904	argininosuccinate synthase 1
Atp2b4	29600	0.988	ATPase, Ca++ transporting, plasma membrane 4
Atp6v1b1	312488	1.172	ATPase, H transporting, lysosomal V1 subunit B1
C5	362119	1.030	complement component 5
Ca2	54231	1.189	carbonic anhydrase 2
Calb1	83839	2.061	calbindin 1
Ccl2	24770	0.949	chemokine (C-C motif) ligand 2
Cd36	29184	-0.734	CD36 molecule (thrombospondin receptor)
Cdh11	84407	1.455	cadherin 11
Cdh6	25409	1.645	cadherin 6
Cdkl1	314198	1.619	cyclin-dependent kinase-like 1 (CDC2-related kinase)
Celsr2	83465	1.430	cadherin, EGF LAG seven-pass G-type receptor 2 (flamingo homolog, Drosophila)
Clmn	299285	1.518	calmin
Cp	24268	1.008	ceruloplasmin (ferroxidase)
Cyp4a1	50549	1.217	cytochrome P450, family 4, subfamily a, polypeptide 1
Cyp4a2	24306	1.320	cytochrome P450, family 4, subfamily a, polypeptide 2
Ddah1	64157	0.785	dimethylarginine dimethylaminohydrolase 1
Ddr1	25678	0.684	discoidin domain receptor tyrosine kinase 1
Dlc1	58834	-0.585	deleted in liver cancer 1
Dll4	311332	-0.695	delta-like 4 (Drosophila)
Efemp1	305604	1.450	EGF-containing fibulin-like extracellular matrix protein 1
Efhd1	501181	1.063	EF-hand domain family, member D1
Egf	25313	2.094	epidermal growth factor
Emx2	499380	0.975	empty spiracles homeobox 2
Epcam	171577	1.332	epithelial cell adhesion molecule
Errfi1	313729	0.944	ERBB receptor feedback inhibitor 1
Eya1	502935	0.935	eyes absent homolog 1 (Drosophila)
F2rl1	116677	0.786	coagulation factor II (thrombin) receptor-like 1
Fam20c	304334	1.312	family with sequence similarity 20, member C
Fam212a	316001	-0.659	family with sequence similarity 212, member A
Fras1	289486	1.604	Fraser syndrome 1
Gpc3	25236	0.778	glypican 3
Gpm6a	306439	0.792	glycoprotein m6a
Gpr56	260326	1.101	G protein-coupled receptor 56
Heyl	313575	0.781	hairy/enhancer-of-split related with YRPW motif-like
Hlx	364069	-0.735	H2.0-like homeobox
Hnf1b	25640	1.786	HNF1 homeobox B
Hsd11b1	25116	0.915	hydroxysteroid 11-beta dehydrogenase 1
Hyal1	367166	-0.632	hyaluronoglucosaminidase 1
Kcnj1	24521	1.384	potassium inwardly-rectifying channel, subfamily J, member 1
Kl	83504	1.003	Klotho
Krt8	25626	1.254	keratin 8

L1cam	50687	1.251	L1 cell adhesion molecule
Lox	24914	1.126	lysyl oxidase
Map7	293016	0.822	microtubule-associated protein 7
Mme	24590	1.139	membrane metallo-endopeptidase
Myh11	24582	0.856	myosin, heavy chain 11, smooth muscle
Nav2	171563	0.816	neuron navigator 2
Ncs1	65153	0.956	neuronal calcium sensor 1
Ndrg1	299923	0.949	N-myc downstream regulated 1
Ndrg2	171114	0.696	N-myc downstream regulated gene 2
Nos1	24598	0.823	nitric oxide synthase 1, neuronal
Pax2	293992	0.697	paired box 2
Pax8	81819	1.552	paired box 8
Pdgfc	79429	1.119	platelet derived growth factor C
Plau	25619	1.654	plasminogen activator, urokinase
Prkcz	25522	0.815	protein kinase C, zeta
Prss8	192107	1.111	protease, serine, 8
Ptprf	360406	1.040	protein tyrosine phosphatase, receptor type, F
Ramp2	58966	-0.645	receptor (G protein-coupled) activity modifying protein 2
Rbp1	25056	1.171	retinol binding protein 1, cellular
Ren	24715	1.544	renin
S1pr1	29733	-0.642	sphingosine-1-phosphate receptor 1
Slc12a1	25065	2.566	solute carrier family 12 (sodium/potassium/chloride transporters), member 1
Slit2	360272	1.160	slit homolog 2 (Drosophila)
Slit3	83467	0.940	slit homolog 3 (Drosophila)
Sostdc1	266803	1.403	sclerostin domain containing 1
Spint1	311331	0.767	serine peptidase inhibitor, Kunitz type 1
Spta1	289257	0.976	spectrin, alpha, erythrocytic 1 (elliptocytosis 2)
Tagln	25123	0.738	transgelin
Tfcp2l1	304741	1.386	transcription factor CP2-like 1
Tfrc	64678	0.754	transferrin receptor
Umod	25128	2.605	uromodulin
